# Exogenous Application of Foliar Salicylic Acid and Propolis Enhances Antioxidant Defenses and Growth Parameters in Tomato Plants

**DOI:** 10.3390/plants10010074

**Published:** 2021-01-01

**Authors:** Nouran Ahmed Abdo Abd El-Hady, Abdelaleim Ismail ElSayed, Sayed Soliman El-saadany, Paola A. Deligios, Luigi Ledda

**Affiliations:** 1 Biochemistry Department, Faculty of Agriculture, Zagazig University, 44519 Zagazig, Egypt; nouran_111ahmed@yahoo.com (N.A.A.A.E.-H.); aleim_77@yahoo.com (A.I.E.); prof.sayedelsaadany@gmail.com (S.S.E.-s.); 2 Department of Agricultural Sciences, University of Sassari, 07100 Sassari, Italy; pdeli@uniss.it; 3 Department of Crop, Food and Environmental Sciences, Marche Polytechnic University, 60131 Ancona, Italy

**Keywords:** *Solanum lycopersicum*, agronomic traits, fruit yield, flavonoids, chlorophyll content, antioxidant enzyme activity

## Abstract

Salicylic acid (SA) and propolis (PR) are known to regulate the physiological process and to have a relevant role in bioactive compounds content. Our experiment was designed to evaluate the effect of SA and PR application on the growth, yield, and quality parameters of tomato grown for the fresh market in field conditions in Egypt. We studied the effect of twelve treatments where SA (0.50, 1.00, 1.50, 2.00, and 2.50 mM) and PR (1, 2, 10, 20, and 100 mg propolis mL^−1^) were applied at increasing doses as a sole agent or combined each other (1.50 mM + 10 mg mL^−1^ for SA and PR, respectively). An untreated control was also considered. Tomato plants treated with SA (0.50, 1.00, and 1.50 mM) showed a significant effect in all traits especially SA1 (0.50 mM) in growth parameters and SA2 (1.00 mM) in pigment and antioxidant content. Propolis foliar application was more effective than SA as it revealed that raising the concentration of aqueous extract enhanced the growth parameters and pigment in tomato. The best result was obtained by the 10 mg mL^−1^ treatment. The effect of propolis on antioxidant enzymes varied as the 10 mg mL^−1^ treatment was effective on peroxidases and superoxide dismutase, while 100 mg mL^−1^ was more effective on catalase. Salicylic acid and propolis have a positive effect on both preserving tomato plants and on nutrient supply, so the mixed intermediate concentration (1.50 mM + 10 mg mL^−1^) is considered very effective and results in an improvement of all plant traits.

## 1. Introduction

Tomato (*Solanum lycopersicum* L.) is considered one of the most significant vegetable crops around the world. In 2017, about 182 million tons of tomatoes were harvested in the world. Furthermore, Egypt ranked as the fifth largest producer with a cultivated area of 182,444 ha and productivity of 40 t ha^−1^ [1], far lower as compared to the yield achieved in developed countries. Tomato fruits are characterized by a great number of high health-promoting bioactive compounds such as phenolic, carotenoids, vitamins, and glycoalkaloids [2]. Being of tropical origin, the plant species is well adapted to almost all climatic regions; however, the abiotic stresses are the main constraints of potential yield and quality of the tomato [3]. Environmental temperatures are rising due to the current global climate change, threatening the agricultural output [4]. High temperature causes different changes in plants such as physiological, morphological, biochemical, which influence their growth and development [5,6,7]. This leads to a reduction in yields of crop species, and therefore has a great effect on global food production [8]. Tomato plants grown under arid and semi-arid environment conditions are exposed to high temperature; this results in a reduced yield in many *Solanum lycopersicum* cultivars [9,10]. In this context, it is strategic to adopt some new methods to increase tomato production and improve the growth in an increasing temperatures scenario [11]. In several harsh environmental conditions such as drought, salt stress, and high temperature, plants endogenous antioxidant networks are not sufficient to protect [12]. Eventually, several reports have highlighted that cultural practices (e.g., appropriate nutrient solution concentration or use of exogenous adjuvants such as plant extracts) might improve the antioxidant composition of fruits or increase plants tolerance to stress due to different factors including salinity, high temperature, and diseases [13,14]. Propolis (PR) is considered a complex mixture consisting of compounds released by bees and derived from plants; therefore, its chemical composition varies due to the geographical and vegetal origins of these resins, as well as bee species [15,16]. Therefore, many studies reported the antimicrobial and antibiotic activities of bee propolis [17,18,19]. Salicylic acid (SA) is a natural growth regulator of vascular plants that impacts different physiological and metabolic processes, e.g., photosynthesis, transpiration, ion uptake, and transportation [20]. Exogenous application of salicylic acid increased the yield of vegetable species by reducing stress-induced growth reduction [21]. It was reported that the growth-promoting effects of SA may be related to changes in the hormonal status [22,23] or by enhancing of photosynthesis, transpiration, and stomatal conductance [23,24], as well as antioxidant enzyme activities and osmoregulation [25]. The ameliorative effects of SA on tomato plants have been well-documented inducing salt, drought, and low temperature tolerance [26,27,28]. To the best of our knowledge, there are no studies that have discussed the effect of propolis and SA together on tomato. Therefore, the purpose of this study was to assess the protective effect of PR and SA on *Solanum lycopersicum* and yield attributes, physio-biochemical attributes, and antioxidant defense system components under natural environmental conditions (i.e., normal climate change conditions).

## 2. Results

### 2.1. Morphological Characterization and Yield

Treatments significantly affected agronomic and morphological characteristics of the crop (Table 1) with respect to the untreated control. The treatment of salicylic acid mixed with propolis showed the highest value for all traits, except for the number of flower cluster and leaf area, as the highest values were observed for PR5 and SA4, respectively. A steadily increasing effect of the propolis treatments has been observed in all traits by applying increasing concentrations. Accordingly, propolis supplied as solely agent at the highest concentration, namely, 100 mg g^−1^ (PR5), proved to be the second most effective treatment (after SA+PR), especially affecting length, height, and number of branches. An opposite trend was recorded for salicylic acid treatments, where we observed a systematically decreased effect by increasing its concentration (Table 1). Summarizing, compared with control (untreated plants), propolis and salicylic acid treatments showed significant variation in plant phenotypic (length, height, branch number, leaf number, flower cluster, and leaf area). Specifically, as the concentration of propolis used in the treatments increased, significant variations were observed for most of the phenotypic traits taken into account, namely, a greater height of the plant, a greater number of flower cluster, and a wider leaf area. By contrast, salicylic acid showed an opposite effect with respect to the increase of tested concentrations. Indeed, treatment SA5 (salicylic acid concentration 2.50 mM) showed a number of leaves and flower clusters per plant, and also a leaf area lower than the untreated control.

The number and the weight of fruits were significantly (*p* < 0.05) influenced by treatments (Table 2), and foliar application of propolis was more effective than salicylic acid. Indeed, the PR5 treatment provided the highest fruits number with 18 fruit plant^−1^, followed by PR4 and SA1. The lowest number of fruit was obtained in the SA5 treatment. The fruit weight was significantly higher in SA1, SA2, and PR5. The highest fruit yield (Table 2) was recorded for the SA+PR treatment with 6.7 kg FW m^−2^. Propolis confirmed to be more effective than SA, as the application of PR5 produced 2.5 kg FW m^−2^ for early yield and 6.3 kg FW m^−2^ for total yield.

### 2.2. Biochemical Compounds Content

The maximum total soluble solids (TSS) was recorded in SA+PR and the lowest amount in SA5 (Table 3). A progressive increase in the total flavonoids and phenolic content was observed at higher concentrations of salicylic acid and propolis. The highest level of total flavonoid and total phenolic was recorded in PR5 treatment. The highest effect on protein content was recorded in SA5 followed by PR5.

### 2.3. Antioxidant Enzyme Activity

**Figure 1:**
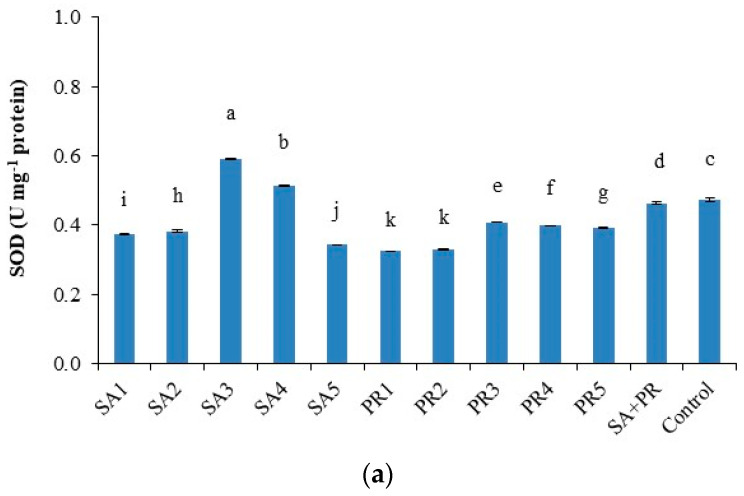

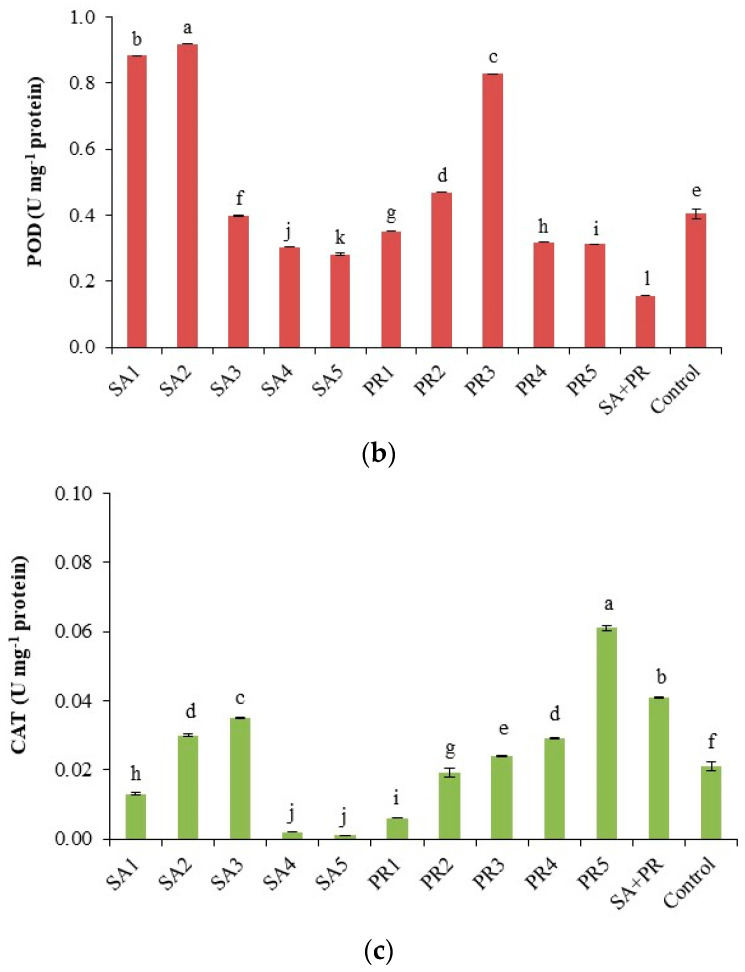
Antioxidant enzyme activities in tomato after treatments. (**a**) Superoxide dismutase, SOD (U mg^−1^ protein); (**b**) peroxidases, POD (U mg^−1^ protein); (**c**) catalase CAT (U mg^−1^ protein). The data presented are the mean of three replicates. Significant differences (*p* < 0.05) among the treatments are indicated by different letters, ± bars indicate standard errors of the mean.

In the range of SA1-SA4, the maximum activity of SOD and POD was observed (Figure 1a,b). Propolis had the greatest antioxidant enzymes activity at PR3 (for POD), even if significantly lower than SA2 and SA1 (Figure 1b). The PR5 treatment and mixed concentration were the most effective in affecting CAT enzyme amount (Figure 1c).

### 2.4. Chlorophyll and Carotenoid Content

**Figure 2:**
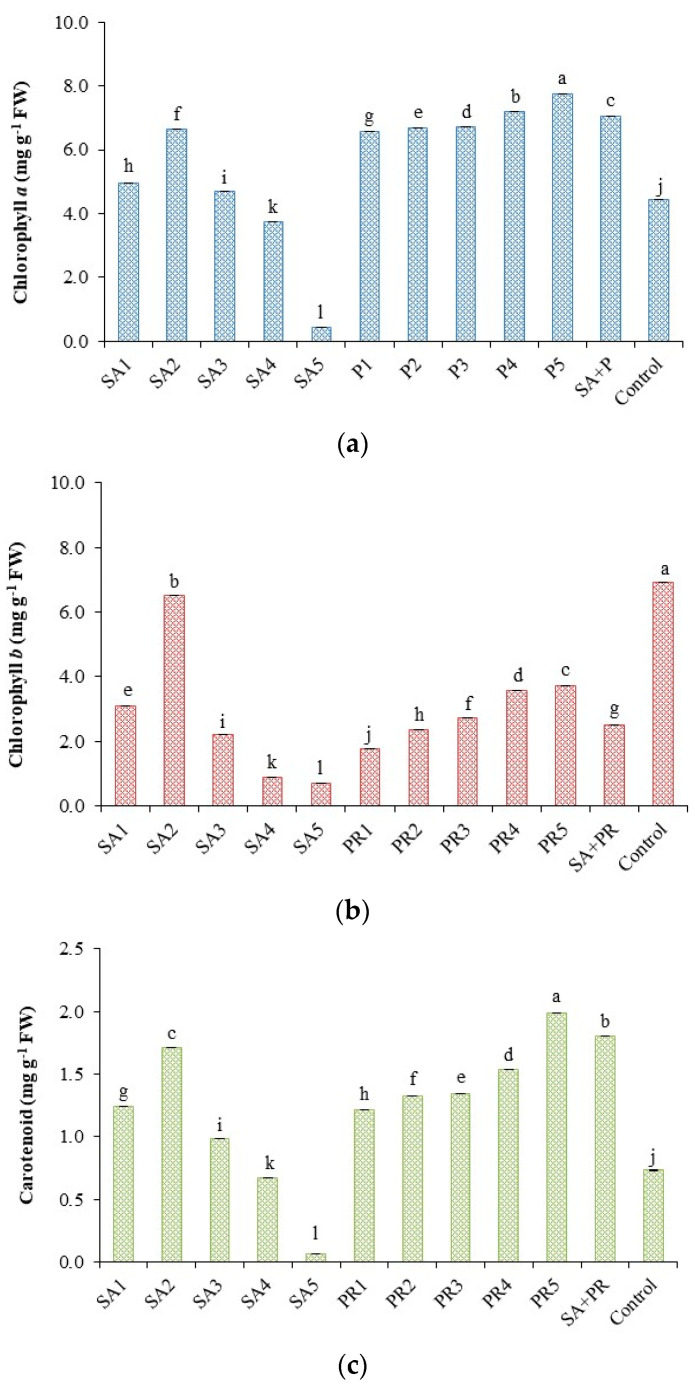
Effect of different concentrations of salicylic acid (SA) and propolis (PR) on (**a**) chlorophyll *a*; (**b**) chlorophyll *b*; and (**c**) carotenoid content in tomato (mg g^−1^ FW). The data presented are the mean of three replicates. Significant differences (*p* < 0.05) among the treatments are indicated by different letters; where visible, ± bars indicate standard errors of the mean.

Application of high concentration of propolis and the mixed treatment SA+PR caused a significant increase in chlorophyll *a*, chlorophyll *b*, and carotenoids (Figure 2). In particular, propolis at the highest concentration determined the maximum chlorophyll *a* (Figure 2a) and carotenoids content (Figure 2c). The control was highest in chlorophyll *b* content, significantly different from SA2 and PR5 (Figure 2b).

## 3. Discussion

### 3.1. Morphological Characterization and Yield

Salicylic acid had a stimulation effect on growth characters of tomato plants and this effect increased by increasing salicylic acid concentration in the range 0.50–2.50 mM as also reported by Yildrim and Dursun [29] who found highest tomato yield at 0.50 mM salicylic acid foliar treatment. Moreover, our results are confirmed by Gharib [30] that investigating the application of SA at low concentration observed a higher photosynthetic activity, which enhanced plant height, number of branches and leaves, as well as leaf area, fresh and dry weight. Our study clarified the enhancing and stimulatory effect of the aqueous extract of propolis as foliar spray on tomato plants. The increase in vegetative growth that we have observed was probably due to its high level of biochemical compounds [31,32]. Indeed, the propolis contains large amounts of antioxidants owing to the extent of flavonoids and other effective compounds that prevent the oxidation and damage of the plant [33] and lead to organize plant growth [34]. Additionally, the effectiveness of propolis may also be related to the extraction method; as Santos [35] reported, the aqueous extract had the highest antioxidant activity because of its highest content of phenolic compounds. In this study, the use of high concentrations of propolis and low concentrations of salicylic acid, or the combination of the two agents (SA+PR treatment), provided the highest yields. The increase of fresh weight that we observed in Table 2 may be due to the promoted availability of micro- and macro-nutrients [36,37]. In our experiment, propolis and salicylic acid have been proved to have a vital role in fasten maturity in tomato plants, and these results are confirmed by other studies [8,29]. Specifically, for propolis, it was argued that it does not increase the early yield directly, but preserves the fruits by forming a protective layer against different types of microbes, especially fungi that affect the fruits of tomatoes greatly and lead to a high loss of young fruits [38,39].

The increasing values of growth parameters of plants treated with propolis could also be attributed to the rise in indoles in these plants that might stimulate an increase in cell division and enlargement [40,41], beside the highest amount of terpenoids that enhance the plant metabolism [42], as well as fresh and dry weight [43,44].

### 3.2. Biochemical Compounds Content

In agreement with our findings, other studies reported that the amount of acidity, TSS, and soluble protein were influenced by SA treatment [45,46]. The explanation is probably due to the fact that SA induces the production of hydrogen peroxide, which stimulates a great activity of phenylalanine ammoniumlyase, responsible for the synthesis of phenolic compounds [47]. The SA plays a vital role in increasing the synthesis of phytochemical compounds and antioxidants as well as activates the secondary metabolism, as reported by Mora-Herrera et al. [48]. Salicylic acid possibly improved protein content by the induction of protein kinase synthesis [18,41,49] whereas through regulation of proline synthesis led to increase the plant defense system and activate adaptive responses. Propolis treatments significantly increased total protein and proline content as observed in Table 3 compared with the control plants as also recorded by El-Yazal working on spinach [50].

### 3.3. Antioxidant Enzyme Activity

SOD, POD, and CAT enzymes protect plants’ cellular activity and they modify scavenger enzymes expression in the cell membrane system against oxidative damage [51,52]. Our findings are confirmed by results of other studies carried out on different species [53,54]. In our study, a significant increase of SOD and POD was observed by SA applications. This was similar to the observed increase in antioxidant enzymes activity in tomato plants recorded by Hayat et al. [55]. According to the propolis, there was not any previous study that indicated the role of foliar application on POD, SOD, and CAT antioxidant enzymes. Our results in Figure 1 showed a significant effect on the concentration of antioxidant enzymes because of foliar application of propolis. This is because propolis is considered natural material rich in bioactive compounds such as polyphenolic compounds, flavones, flavonones, as well as phenolic and antioxidant enzymes [56]. Total polyphenol and flavonoid contents are investigated to be the most effective antioxidant in propolis [57].

### 3.4. Chlorophyll and Carotenoid Content

Leaf pigments concentration (chlorophyll *a*, chlorophyll *b*, and total carotenoids) raised by applying propolis extract as a foliar treatment was also found by other studies [58]. The increment of leaf pigments concentration of propolis treatments might be attributed to the rise in their treatments hormones and enhances mineral absorption, i.e., (Fe and Me), which are required for chlorophyll synthesis [59,60]. The mixed concentration of (SA+PR) showed the most significant value in most traits. This is due to the effect of two intermediate concentrations of two bioactive substances, one of which positively affects growth factors and the other affects the content of biochemical and photosynthesis of plants. This may be regarded as the first result of its kind, which proves the effectiveness of two natural vital substances, one of them from a botanical source and the other from the secondary products of bees.

## 4. Materials and Methods

### 4.1. Experimental Conditions

**Figure 3:**
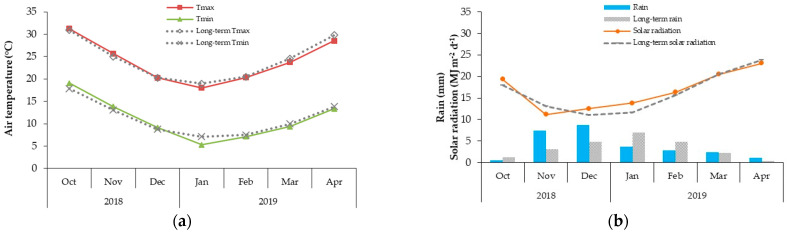
Seasonal time series for (**a**) air temperatures (°C); and (**b**) rain (mm) and solar radiation (MJ m^−2^ d^−1^) over the Sharkia governorate region (1989–2012) and comparison with the weather trend occurred during the 2018–2019 growing season.

The field trial was conducted in the new Salhia in Sharkia governorate (72° 32′ E; 23° 3′ N), Egypt, during the October 2018–2019 growing season. The soil of the area is clay-loam with 8.1 pH, 0.92% soil organic matter content, and 0.01% N. In this location, usually June is a rainless month, whereas most rainfall occurs in December (average monthly rainfall: 7 mm). Yearly, the hottest month is August (average monthly temperature: 26.8 °C) [61] (Figure 3).

### 4.2. Plant Material and Experimental Design

To improve physical and chemical properties of tomato plants, two different stimulants were tested sole or in combination: salicylic acid (SA; 2-hydroxybenzoic acid; Sigma Chemical Co., Gillingham, UK) and propolis (as an organic biostimulant) (taken from the apiary of Beekeeping Research Section, Plant Protection Research Institute, Agriculture Research Centre at Dokki, Giza, Egypt). The chemical description of the propolis used in this study is presented in Table 4. A total of twelve treatments were tested. The tomato hybrid Al-Quds E448 (Ministry of Agriculture-Tadress Lyon Company, Cairo, Egypt) was considered for the experiment. The experiment was organized in a completely randomized block design with three replications. Each experimental unit size was 20 m^2^ with five rows (150 plants per plot), 9 m in length, and 50-wide row spacing.

### 4.3. Field Management and Treatment Description

The tomato seedlings were transplanted on 10 October 2018, and whole seedling samples were taken before the experiment began. During the experiment, 100 g natural organic fertilizer (Cow manure, Hebei Shuanglian Biological Technology Co., Hebei, China), 100 g chicken manure compost (Hebei Shuanglian Biological Technology Co., Hebei, China), 1000 g agricultural sulfur (Agriculture soreil, Kafr El Zayat Pesticides and Chemicals Co., Kafr El Zayat, Egypt), 500 g of N (Ammonia sulphate, SEMADCO Co., Suez, Egypt), 75 g of P_2_O_5_ (Superphosphate, Suez Company For Fertilizer Production, Suez, Egypt), and 500 g of K_2_O (Potassium sulphate, Suez Company For Fertilizer Production, Suez, Egypt) were supplied by fertigation. No insecticides and fungicides were used during the experiment. Weeds were controlled manually. Salicylic acid was initially dissolved in 100 µL dimethyl sulfoxide and concentrations of 0.50 (SA1 treatment), 1.00 (SA2 treatment), 1.50 (SA3 treatment), 2.00 (SA4 treatment), and 2.50 mM (SA5 treatment) at pH 6.0–6.5; the solutions were completed with distilled water containing 0.02% Tween 20 (Polyoxyethylenesorbitan monolaurate, Sigma Chemicals, Gillingham, UK) [62]. The first SA treatment occurred after 20 days when the young plants had 2–3 true leaves. Leaves, both in the lower and upper surfaces [63], were sprayed with the SA solutions until dripping with a held atomizer. Before utilization, 50 g of propolis was freeze-dried for three hours, suspended and extracted with 50 mL of ethanol (70%), and kept on a shaker at 150 rpm for two days at 26 °C. Then, the extract was centrifuged at 28,000× *g* for 30 min, and the supernatant was collected and evaporated at room temperature (25 °C) for 3 days; thus, the remaining resin was collected for further testing [64]. Dilutions of 1:10, 1:50, 1:100, 1:500, and 1:1000 were prepared with the final concentrations of 1 (PR1 treatment), 2 (PR2 treatment), 10 (PR3 treatment), 20 (PR4 treatment), and 100 (PR5 treatment) mg propolis mL^−1^ distilled water, respectively, and then temporarily stored at room temperature. The initial foliar propolis treatment occurred after 20 days when the seedlings had 2–3 true leaves. The propolis was sprayed with the solutions until dripping with a held atomizer. Plants treated with water were used as untreated control (Control treatment). An additional treatment (SA+PR) mixing salicylic acid (1.50 mM) with propolis (10 mg propolis mL^−1^) was tested. Treatments were applied at a 15-day time interval, and after 10 days from the last treatment, vegetative samples were taken.

### 4.4. Growth and Quality Monitoring and Analysis

Sixty days after transplanting, four plants were harvested for each replication, and data on plant growth variables were collected (e.g., plant length and height; branches and leaves number; plant leaf area, the number of flower clusters and fruits, the unit fruit weight, early and total yield). The yield of the first three pickings (25% of pickings number) was calculated as the early yield. At the end of the crop cycle, the total production was calculated. Plant height was measured from the ground level to the apical meristem of the main stem and plant length from the roots to the apical meristem of the main stem carrying the longest leaf. Plant dry weight was mathematically obtained by summing leaf and stem dry weight; whereas the number of leaves per plant was determined by counting all leaves of the plant including new tips and sprouts. Leaf area per plant was calculated utilizing the leaf area–leaf weight relationship as described by Taha and Osman [65]. Leaf area per plant (cm^2^) was obtained utilizing the formula in which the total leaf dry weight (g) is LDW, the disks dry weight (g) is DDW, and the area of disks (cm^2^) is DA:

$$\mathrm{Leaf}\;\mathrm{area}/\mathrm{plant}=\frac{\mathrm{LDW}}{\mathrm{DDW}}\times \mathrm{DA}.$$

The total yield for each treatment was calculated by weighing the fruits picked in each replication and converting the weight into kg per m^2^. In addition, the average percentage of dry matter and total soluble solids (TSS %) were determined as described in AOAC official methods [66] as indictors of fruit quality.

### 4.5. Leaf Pigments

Leaf pigments contents, namely, chlorophyll *a*, *b* and carotenoid (mg g^−1^ fresh weight) were quantitatively determined and calculated as described by Lichtenthaler [67].

### 4.6. Biochemical Measurements

Total phenolic compounds (TPCs) were estimated (mg g^−1^ FW) by the Folin–Ciocalteu reagent as reported by Singleton et al. [68]. Total flavonoids (TFs) were established (mg g^−1^ FW) according to the protocol of Ordonez et al. [69]. For the quantitatively measurements of antioxidant enzymes activities, the samples of leaves were milled and turned into fine powder in liquid nitrogen and extracted with 10 mL of 50 mM phosphate buffer, pH 7.0. The homogenate was centrifuged at 20,000× *g* for 30 min at 4 °C. In order to measure antioxidant enzyme activities, the supernatant was then filtered and used. Total soluble protein content was measured by utilizing the Bradford procedure [70]. Superoxide dismutase (SOD) activity assayed as reported in Giannopolitis and Ries [71]. Catalase (CAT) activity assayed as suggested by Aebi (1984) [72]. In tomato leaves, peroxidase (POD) activity was determined utilizing the procedure by Thomas et al. [73]. All solvents used in this work were obtained from different companies. Gallic acid, quercetin, DPPH˙, and substrates were purchased by Sigma Chemical Co., Gillingham, UK.

### 4.7. Statistical Analysis

Data presented as the mean of three independent determinations. One-way ANOVA was used to evaluate the impact of treatments on all parameters with the Duncan’s multiple range test. The differences between the means were considered significant at *p* ≤ 0.05. All analyses were performed using the SAS statistical software (SAS v. 9.2, SAS Institute, Cary, NC, USA).

## 5. Conclusions

The utilization of salicylic acid as a foliar application on tomato plants had a great effect as a growth regulator probably because takes part in the adjustment of plant physiological processes, especially when it is used at small concentrations from 0.50 mM to 1.50 mM. On the other hand, propolis has not only increased the total plant performance, with regard to development and production, but it has also shown a relevant effect on the biochemistry of tomato plants especially in the effect on antioxidant enzymes. The mixed concentration of propolis and SA has been the most effective treatment to enhance tomato plants with physiological properties and nutrient elements and to adapt open field tomato cropping to climate change temperature trends.

## Figures and Tables

**Table 1:** Effect of treatments on the main morphological and agronomic parameters of tomato during the 2018–2019 growing season.

Treatments	Length (cm)	Height (cm)	Branch (no. plant^−1^)	Leaf (no. plant^−1^)	Flower Cluster (no. plant^−1^)	Leaf Area (cm^2^ plant^−1^)
SA1 ^†^	90.0 cd	87.7 cd	8.0 ab	55.3 ab	10.0 cdef	753.6 cde
SA2	88.3 de	85.7 d	7.0 abc	49.3 abcd	9.7 cdef	942.0 cde
SA3	84.3 de	79.7 de	7.0 abc	47.0 cde	8.6 def	1413 abc
SA4	80.0 de	78.3 de	5.3 bcd	39.7 efg	8.0 ef	2025 a
SA5	78.0 e	73.3 e	5.0 cd	33.7 g	7.0 f	134.3 e
PR1 ^‡^	90.0 cd	86.3 d	7.0 abc	35.0 fg	11.6 bcde	1077 bcd
PR2	99.7 bc	96.0 bc	7.0 abc	45.6 cde	12.3 bcd	1119 bcd
PR3	102.3 b	97.3 b	7.3 abc	46.6 cde	13.6 abc	1830 ab
PR4	102.7 b	99.0 b	7.7 abc	47.3 bcde	15.3 ab	752.3 cde
PR5	104.7 b	100.6 b	8.3 a	52.3 abc	17.0 a	1123 bcd
SA+PR ^§^	116.3 a	113.6 a	8.7 a	56.0 a	15.3 ab	1178 bcd
Control	53.3 f	48.3 f	3.7 d	42.6 def	9.0 def	434.8 de

Mean values in each column followed with different letters indicate significant differences at *p* < 0.05 level. ^†^ SA1-SA5: salicylic acid treatment at 0.50, 1.00, 1.50, 2.00, and 2.50 mM, respectively. ^‡^ PR1-PR5: propolis treatment at 1, 2, 10, 20, and 100 mg propolis mL^−1^ concentration, respectively. ^§^ SA+PR: 1.50 mM + 10 mg mL^−1^ for salicylic acid and propolis, respectively.

**Table 2:** Effect of treatments on the yield parameters of tomato plants during the 2018–2019 growing season.

Treatments	Fruit (no. plant^−1^)	Fruit Weight (g unit^−1^)	Early Yield (kg FW m^−2^)	Total Yield (kg FW m^−2^)
SA1 ^†^	15.7 abc	95.5 a	1.3 cde	5.2 bc
SA2	14.0 bcd	95.3 a	1.2 de	5.0 bcde
SA3	13.7 bcd	82.8 d	1.0 e	4.8 cdef
SA4	12.3 cd	80.2 d	1.0 e	4.1 ef
SA5	12.0 d	72.3 e	0.9 e	4.0 f
PR1 ^‡^	13.3 bcd	73.0 e	1.7 bcd	4.3 cdef
PR2	14.0 bcd	80.7 d	1.8 bc	5.0 bcde
PR3	15.7 abc	83.7 d	2.1 ab	5.1 bcd
PR4	16.7 ab	88.0 c	2.2 ab	5.8 ab
PR5	18.0 a	93.3 ab	2.5 a	6.3 a
SA+PR ^§^	16.0 ab	91.3 bc	2.6 a	6.7 a
Control	11.0 d	66.2 f	1.0 e	4.2 def

Mean values in each column followed with different letters indicate significant differences at *p* < 0.05 level. ^†^ SA1-SA5: salicylic acid treatment at 0.50, 1.00, 1.50, 2.00, and 2.50 mM, respectively. ^‡^ PR1-PR5: propolis treatment at 1, 2, 10, 20, and 100 mg propolis mL^−1^ concentration, respectively. ^§^ SA+PR: 1.50 mM + 10 mg mL^−1^ for salicylic acid and propolis, respectively.

**Table 3:** Effect of treatments on the biochemical changes of tomato plant leaves during the 2018–2019 growing season.

Treatments	Total Soluble Solids (%)	Total Flavonoids (mg g^−1^ FW)	Total Phenolic (mg g^−1^ FW)	Protein Content (mg g^−1^ FW)	Proline (mg g^−1^ FW)
SA1 ^†^	4.63 c	130.80 k	136.73 j	43.55 e	0.107 k
SA2	4.66 bc	169.76 j	142.03 i	46.10 de	0.114 i
SA3	4.57 c	213.40 h	154.90 h	51.45 bcde	0.117 h
SA4	4.33 d	221.00 f	155.23 g	57.95 bc	0.319 d
SA5	3.90 f	222.60 d	161.26 d	74.75 a	0.649 a
PR1 ^‡^	3.30 g	211.10 i	159.60 f	47.45 cde	0.111 j
PR2	4.13 e	221.00 f	160.06 e	49.90 bcde	0.111 j
PR3	4.60 c	221.80 e	167.46 c	54.75 bcde	0.141 f
PR4	4.80 b	222.80 c	174.80 b	56.95 bcd	0.145 e
PR5	5.20 a	225.90 a	193.20 a	60.20 b	0.394 c
SA+PR ^§^	5.27 a	223.76 b	131.63 k	50.95 bcde	0.516 b
Control	4.33 d	220.03 g	128.73 l	48.95 bcde	0.127 g

Mean values in each column followed with different letters indicate significant differences at *p* < 0.05 level. ^†^ SA1-SA5: salicylic acid treatment at 0.50, 1.00, 1.50, 2.00, and 2.50 mM, respectively. ^‡^ PR1-PR5: propolis treatment at 1, 2, 10, 20, and 100 mg propolis mL^−1^ concentration, respectively. ^§^ SA+PR: 1.50 mM + 10 mg mL^−1^ for salicylic acid and propolis, respectively.

**Table 4:** Chemical description of the Egyptian propolis (honey bee species *Apis mellifera lamarckii*).

Parameter	Value
Moisture (%)	7.05
Proteins (%)	11.03
Fats (%)	23.12
Fibers (%)	51.02
Carbohydrates (%)	6.02
Ash (%)	2.11
Resin (%)	57.92
Insoluble matter (%)	40.91
Volatile substances (%)	3.33
Total phenolic content (mg GAE g^−1^ sample DW)	253.70
Total flavonoid content (mg quercetin g^−1^ sample DW)	76.77
Total alkaloid (g 100 g^−1^ FW)	5.42

## Data Availability

The data presented in this study are available on reasonable request from the corresponding author. The datasets generated and analyzed during the current study are not publicly available due to a further related paper that deals with ecotypes used in the experiment and the genotype × environment interaction.
